# Characterization of the Immune Infiltration Landscape and Identification of Prognostic Biomarkers for Esophageal Cancer

**DOI:** 10.1007/s12033-022-00526-9

**Published:** 2022-07-03

**Authors:** Yuanmei Chen, Xinyi Huang, Lin Chen, Guibin Weng, Zhengrong Huang, Yangfan Zhang, Tianya Xiao, Junqiang Chen, Kunshou Zhu, Yuanji Xu

**Affiliations:** 1 Department of Thoracic Surgery, Fujian Medical University Cancer Hospital, Fujian Cancer Hospital, Fuzhou, Fujian China; 2 Department of Radiation Oncology, Fujian Medical University Cancer Hospital, Fujian Cancer Hospital, Fujian, China; 3 Department of Integrative Traditional Chinese and Western Medicine, Fujian Medical University Cancer Hospital, Fujian Cancer Hospital, Fuzhou, Fujian China; 4 Fujian Key Laboratory of Innate Immune Biology, Biomedical Research Center of South China, Fujian Normal University Qishan Campus, Fuzhou, Fujian China

**Keywords:** Esophageal cancer (ESCA), Immune cell infiltration (ICI), Prognosis, Immunotherapeutic responses, Biomarker

## Abstract

Immunotherapy is an effective treatment for esophageal cancer (ESCA) patients. However, there are no dependable markers for predicting prognosis and immunotherapy responses in ESCA. Our study aims to explore immune gene prognostic models and markers in ESCA as well as predictors for immunotherapy. The expression profiles of ESCA were obtained from The Cancer Genome Atlas (TCGA), the Gene Expression Omnibus (GEO), and International Cancer Genome Consortium (ICGC) databases. Cox regression analysis was performed to construct an immune gene prognostic model. ESCA was grouped into three immune cell infiltration (ICI) clusters by CIBERSORT algorithm. The immunotherapy response of patients in different ICI score clusters was also compared. The copy number variations, somatic mutations, and single nucleotide polymorphisms were analyzed. Enrichment analyses were also performed. An immune gene prognostic model was successfully constructed. The ICI score may be used as a predictor independent of tumor mutation burden. Enrichment analyses showed that the differentially expressed genes were mostly enriched in microvillus and the KRAS and IL6/JAK/STAT3 pathways. The top eight genes with the highest mutation frequencies in ESCA were identified and all related to the prognosis of ESCA patients. Our study established an effective immune gene prognostic model and identified markers for predicting the prognosis and immunotherapy response of ESCA patients.

**Supplementary Information:**

The online version contains supplementary material available at 10.1007/s12033-022-00526-9.

## Introduction

Esophageal cancer (ESCA) ranks as the seventh most common cancer and the sixth most common cause of cancer-related death worldwide [1]. Approximately, 508,585 cancer-related deaths of ESCA were reported in 2018 [1]. Surgical removal is the conventional treatment for most types of ESCA. However, many patients still show local recurrence or distant metastasis within a short time after surgery [2, 3]. Although surgery, radiotherapy, and chemotherapy have improved the survival rate of ESCA patients, the prognosis of ESCA remains poor, and the current 5-year overall survival (OS) rate is only 15%–25% [4]. Currently, the prognosis of ESCA patients is mainly determined by postoperative pathological features, which relies on the TNM staging system. However, because of problems such as stage migration, the current TNM staging system is not very accurate [5]. Therefore, the identification of accurate prognostic models and markers is urgently required to improve the prognosis of ESCA patients and develop individualized therapies.

Immunotherapy is a critical therapy for cancer treatment that activates the immune system to target cancer cells, thus enhancing the autoimmune function to eliminate tumors [6]. Some patients can achieve complete remission after immunotherapy [7]. However, immunotherapy only benefits a small number of patients [8]. Immunotherapy in ESCA patients often causes different therapeutic effects, partly because of the lack of dependable markers to predict therapeutic response [9]. Therefore, identifying new markers that predict immunotherapy response is critical to determine the subgroup of ESCA patients that may benefit from these treatments.

One of the mechanisms by which tumors escape immune response is by inhibiting infiltration of immune cells. Increasing evidence has demonstrated that the tumor microenvironment (TME), which includes tumor-infiltrating immune cells (TIICs), plays a vital role in tumor immune evasion and cancer cell proliferation, invasion, and metastasis [10]. The selective enhancement of anti-tumor immunity in the TME but not in non-tumor tissues enables highly effective tumor immunotherapy without side effects [11]. Better understanding of the interactions between tumor cells and the TME and the mechanisms underlying how these promote the occurrence and development of ESCA could offer novel insights into the future treatment strategies. To date, the broad prospects of immune cell infiltration (ICI) in the TME in ESCA have not been clarified.

The purpose of our study was to explore the immune infiltration landscape and potential prognostic biomarkers in ESCA. In our study, we analyzed the differentially expressed immune-related genes between normal esophageal and ESCA tumor samples in The Cancer Genome Analysis (TCGA) cohort. A prognostic model was constructed, and survival analysis and risk assessment were performed. The CIBERSORT and ESTIMATE algorithms were used to analyze the gene expressions of ESCA and normal samples, and an overview of the immunity in ESCA was obtained. We further divided ESCA into three subtypes based on ICI patterns. Moreover, analyses of TCGA-ESCA tumor sample copy number and somatic variations were conducted. Finally, single nucleotide polymorphism (SNP) analyses of the International Cancer Genome Consortium (ICGC) database were performed, including ICGC database from China (ICGC-CN) and ICGC database from the United Kingdom (ICGC-UK). We established a prognostic model and ICI scores, which may predict the prognosis and the response to immunotherapy of ESCA patients.

## Materials and Methods

### Downloading and Processing of Sample Source Data

The expression profiles of ESCA were obtained from four independent cohorts, including TCGA-ESCA, GSE106185, ICGC-CN, and ICGC-UK. Only data from ESCA tumor samples were obtained. The fragments per kilobase million (FPKM) value of TCGA-ESCA expression profile was obtained using the TCGAbiolinks package [12], and the annotation file of the GENCODE27 version was used to convert FPKM to transcripts per million. Only protein-encoding transcripts were considered. Clinical and survival information of ESCA patients in TCGA was extracted from pan-cancer data. The clinical information included age, sex, tumor stage, and ESCA subtypes defined by TCGA. For evaluation of survival, only overall survival (OS) was considered. The copy number variation (CNV) data of TCGA-ESCA cohort were downloaded through Firehose.

### Establishment of the Immune Gene Prognostic Model

Analysis of differentially expressed genes (DEGs) between 11 normal tissues and 141 tumor tissues in TCGA-ESCA cohort was conducted, and DEGs were visualized by heatmaps and volcano plots. To construct an immune-related prognosis model, the immune-related gene sets were obtained from the IMMPORT network platform. Differential analysis of genes in TCGA-ESCA database was performed, and DEGs were visualized by heatmaps and volcano plots. A genetic model related to the prognosis of ESCA was built by univariate and multivariate Cox regression analyses, and survival analysis and risk assessment were performed.

### Consensus Clustering of TIICs

CIBERSORT [13] is an algorithm that can be applied to describe the cell composition of samples through gene expression values in cancers. The CIBERSORT package and LM22 were used to calculate the infiltration of 22 types of immune cells in 141 ESCA samples. The immune and stromal content (immune and stromal scores) of each ESCA sample were evaluated by the ESTIMATE algorithm. Based on the immune infiltration patterns of ESCA samples, unsupervised hierarchical clustering was conducted using the "ConsensusClusterPlus" package [14]. The classification was repeated 1000 times to ensure stability.

### Analysis of DEGs Related to ICI Phenotype

Patients were classified into ICI clusters and genes related to the ICI pattern were examined. The limma package was applied for differential analysis. A false discovery rate (FDR) less than 0.05 and |fold change| more than 1.5 were regarded as the threshold to determine the DEGs between ICI subtypes.

### Dimensionality Reduction and Generation of ICI Score

Unsupervised clustering was conducted to group patients in TCGA-ESCA cohort based on DEGs. The DEGs that were positively and negatively associated with clustering features were called ICI gene features A and B, respectively. The dimensionality of the gene signatures A and B was reduced by the Boruta algorithm, and principal component analysis was used to extract the principal component 1 as the signature score. The final signatures A and B were obtained.

### Gene Ontology (GO) and Kyoto Encyclopedia of Genes and Genomes (KEGG) Functional Enrichment Analysis

GO and KEGG enrichment of signatures A and B were analyzed to explore their possible functions. The clusterProfiler package [15] was used for enrichment analyses, and the outcomes were displayed in bubble charts.

### Gene Set Enrichment Analysis (GSEA) and Gene Set Variation Analysis (GSVA)

Differential expression analysis of the high and low ICI score groups was conducted by limma [16]. A pre-ranked gene list was conducted based on log2FC, and GSEA enrichment analysis was performed (h.all.v7.2.symbol.gmt was used as background set). GSVA can estimate the scores of certain pathways or signatures based on transcriptome data [17]. The high and low ICI score groups were also used to analyze the msigdb.v7.0.symbols gene set and c7.all.v7.0.symbols immune-related gene set, and the GSVA package was used to explore the differences between different samples in these gene sets. The Limma package was used to conduct a differential analysis of the metabolic score. Characteristics that accorded with |log2FC |> 0.2, and adj.*P* < 0.05 were defined as differential expression characteristics. GSVA enrichment analysis can convert hundreds of biological function annotations into a new expression matrix. Here, the GSVA value of each sample was enriched through each specific pathway.

### ICI Score and Immunotherapy Response

Two independent data sets (IMvigor210 and TCGA-ESCA) were obtained for analysis to identify the predictive value of ICI scores for the response to immunotherapy. In the IMvigor210 cohort (cds data), the ICI score was calculated based on the principal component scores of signatures A and B, and the high and low ICI groups were distinguished accordingly.

### Collection and Analyses of CNV and Somatic Mutation Data

To analyze the CNV mutations of patients in TCGA-ESCA database, the mutation data were obtained from TCGA-ESCA. First, the somatic mutation differences between TCGA-ESCA normal and tumor samples were analyzed. The genes and mutation frequencies that mutated on each chromosome were analyzed; these are presented in circle plots. The differential genes with significant correlation were identified by comparing the correlation between CNV and mRNA expressions. These genes may be the driving genes regulating the CNVs of ESCA. GO enrichment analysis was then performed on the identified genes.

GISTIC2.0 analysis of the downloaded CNV fragment was performed by GenePattern5 [18] using the default settings. Genomic regions that are notably amplified or deleted in a group of samples can be identified by GISTIC. Each aberration was allocated a G score, in which the magnitude of the aberration and its frequency in the sample were taken into account. For each important area within the threshold, a "peak" is generated, which represents the part with the highest variation amplitude and frequency in the abnormal area. In addition, a leave-one-out algorithm was used to determine the "broad peak" to take the marginal error in one single sample into account. Each significantly amplified or missing sample was identified, and the genes found in each "broad peak" region were listed. To identify the mutation load of ESCA, the total number of non-synonymous mutations in ESCA was calculated. The high or low ICI score was used to evaluate the somatic changes in ESCA driver genes. The "maftools" package [19] was applied to explore and visualize ESCA driver genes.

### SNP Mutation Analysis in the ICGC Database

The ICGC database includes data of 50 cancer types (or subtypes) regarding abnormal gene expression, somatic mutations, and clinical data. The ICGC includes 89 projects in 17 administrative regions in Asia, Australia, Europe, North, and South America and includes 25,000 tumor genomes. Two datasets of ESCA in the ICGC database were examined, including ICGC-CN and ICGC-UK. Gene mutation analysis was performed, and a waterfall chart was drawn through the mutation matrix. Mutation site analysis was conducted, and survival analysis was conducted based on the top four mutated genes with the most significant differences. Mutations that were significantly related to the survival and prognosis of ESCA were obtained.

### Statistical Analysis

Data processes and analyses were performed using Excel (Microsoft) and R software (version 4.0.2). To compare two groups of continuous variables, the statistical significance of normally distributed variables was analyzed by independent Student's t test, and the difference between non-normally distributed variables was analyzed by the Mann–Whitney U test (Wilcoxon rank-sum test). The Kruskal–Wallis test was conducted to compare more than two groups, while the Wilcoxon test was applied in the comparison of two groups. The X-tile software was used to test the possible cut points iteratively. The cut-off point with the largest rank statistic was selected, and patients in each data set were divided into two categories to reduce the calculation batch effect. In each data set, a Kaplan–Meier plotter was applied to generate survival curves for the subgroups. The log-rank test was applied to evaluate whether the differences were statistically significant. The chi-square test was applied to estimate the correlation mutation frequency between ICI score subgroups and somatic cells, and Spearman analysis was conducted to calculate the correlation coefficient. A two-tailed p value < 0.05 was regarded as statistically significant.

## Results

### Construction of an Immune Gene Prognostic Model for ESCA

**Fig. 1:**
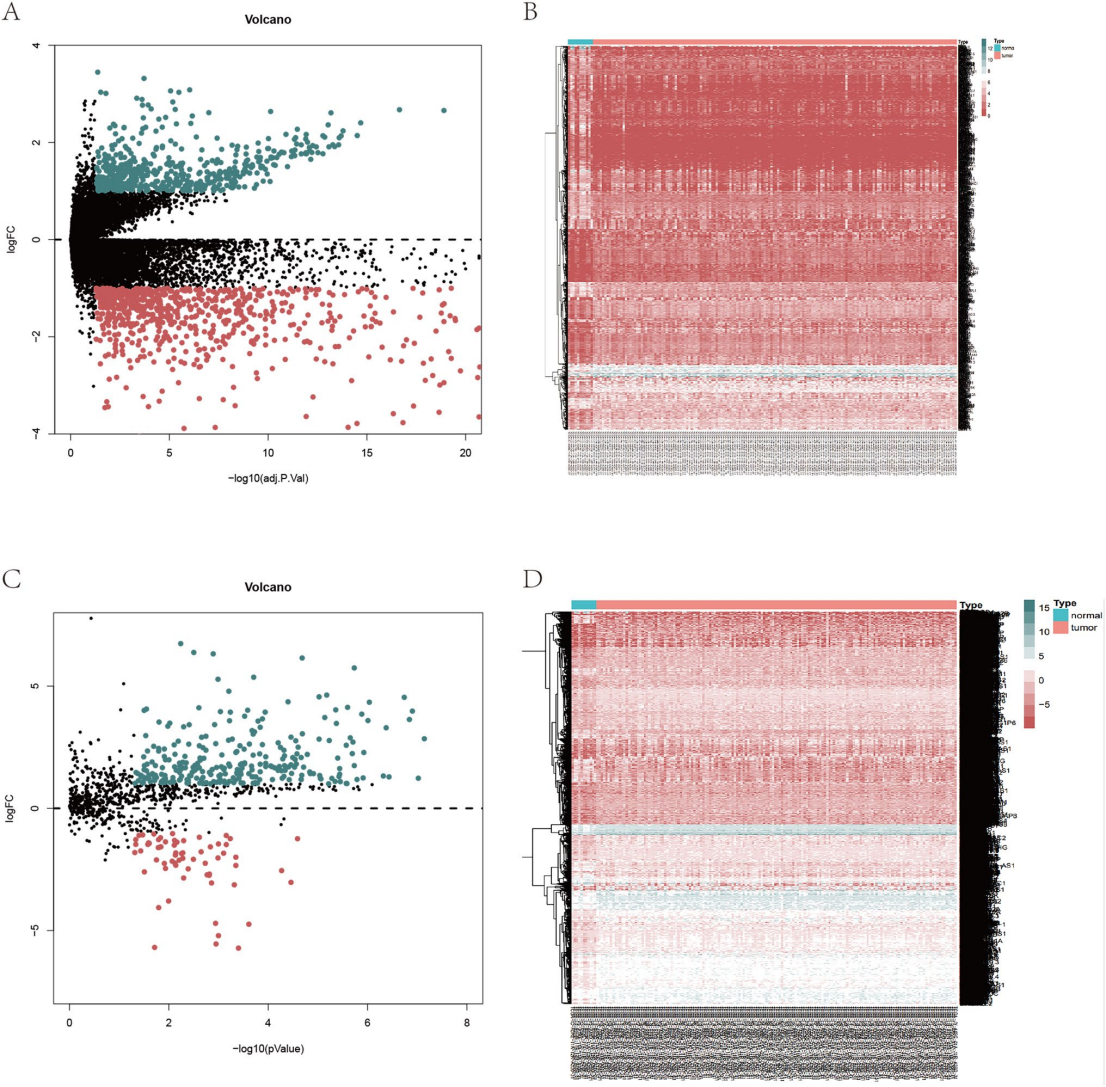
Differential analysis of TCGA-ESCA cohort. Volcano plots of differential genes (**A**) and immune-related differential genes (**C**) between normal and tumor samples in the TCGA-ESCA cohort. The green dots represent significantly up-regulated genes in the tumor samples, the red dots represent significantly down-regulated genes, and the black dots indicate genes with no significant difference in the expression. Heatmaps of differential genes (**B**) and immune-related differential genes (**D**) between normal and tumor samples in the TCGA-ESCA cohort. X-axis, the sample label; y-axis, the differential expression genes

**Fig. 2:**
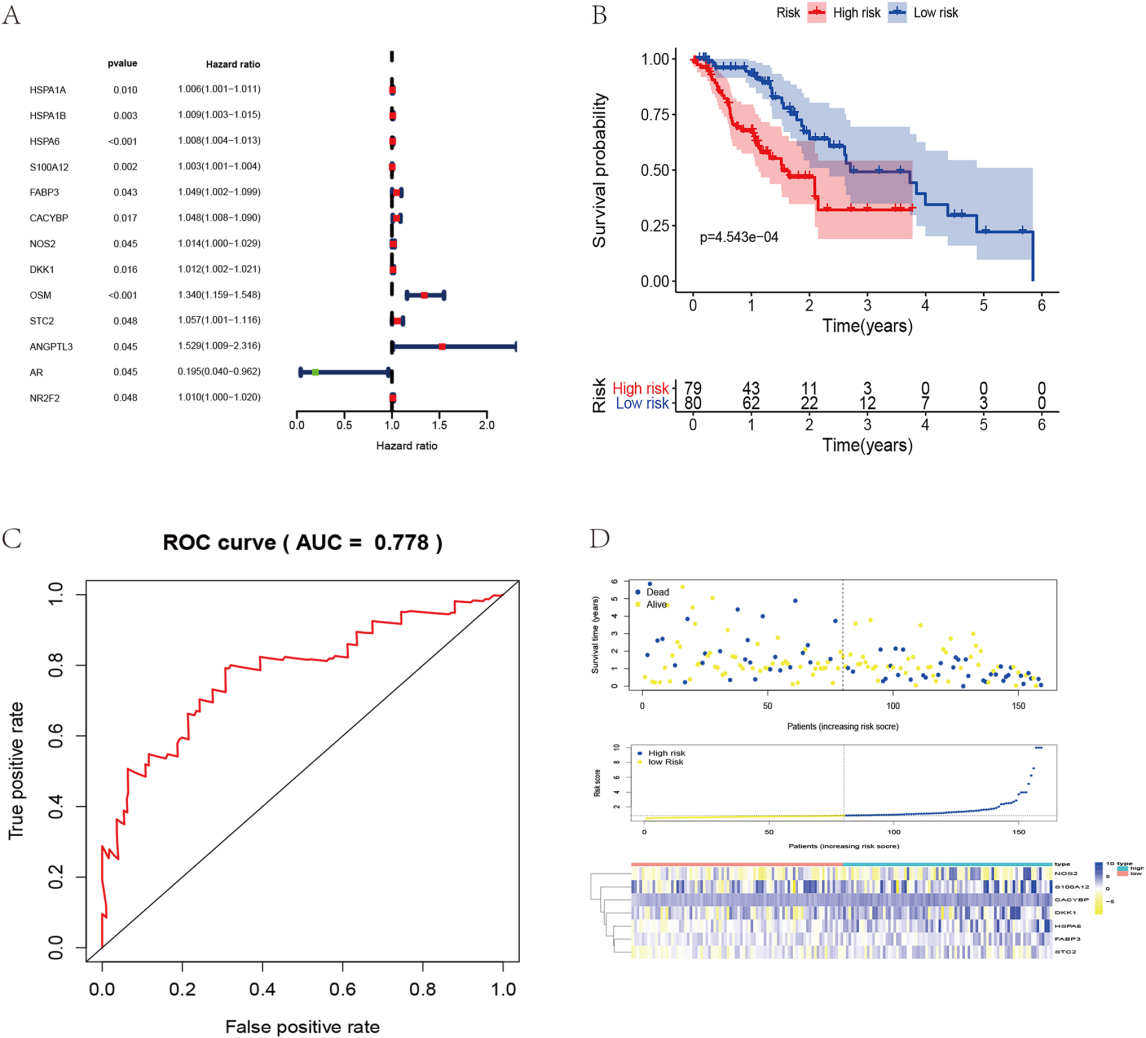
The prognosis of immune genes in TCGA-ESCA cohort and construction of Cox risk regression model. **A** Prognostic value of immune genes in the prognostic model obtained using univariate Cox analysis. **B** Survival analyses of high- and low-risk patients based on the immune gene prognostic model, the effect of immune gene prognostic model on the overall survival (OS) of ESCA was statistically significant. **C** Receiver operating characteristic (ROC) curve of the immune gene prognostic model in predicting the survival prognosis of ESCA. **D** The survival status (top), the risk curve (middle), and risk heat map (bottom) of the immune gene prognostic model set

We analyzed the DEGs between normal esophageal samples and ESCA tumor samples in TCGA, and the DEGs are displayed with heatmaps and volcano plots in Fig. 1A and 1B. Differential analysis of immune-related genes in TCGA-ESCA database was conducted to determine an immune-related prognostic model. The immune-related DEGs were also visualized by heatmaps and volcano plots (Fig. 1C–D). A immune gene prognostic model related to ESCA prognosis was constructed through univariate (Fig. 2A) and multivariate Cox regression analyses (Table 1). Survival analysis (Fig. 2B) and risk assessment (Fig. 2C–D) using the model were performed. Survival analysis suggested a poor prognosis in the high-risk group of the immune gene model (*HSPA6, S100A12, FABP3, CACYBP, NOS2, DKK1,* and *STC2*). ROC curve analysis was conducted to verify the immune gene model prediction. The AUC was 0.778, which proved that the prediction accuracy of the prognostic model was strong.

**Table 1:** Multivariate Cox regression analysis risk model

ID	Coef	HR	HR.95L	HR.95H	P-value
HSPA6	0.0058	1.0058	1.0013	1.0103	0.0112
S100A12	0.0024	1.0024	1.0011	1.0037	0.0003
FABP3	0.0456	1.0466	1.0133	1.0811	0.0058
CACYBP	0.0307	1.0312	1.0009	1.0624	0.0435
NOS2	0.0172	1.0174	1.0069	1.0279	0.0011
DKK1	0.0081	1.0081	1.0005	1.0158	0.0373
STC2	0.0385	1.0392	0.9996	1.0804	0.0521

### The Immune and Stromal Score and ICI Subtype Clustering

**Fig. 3:**
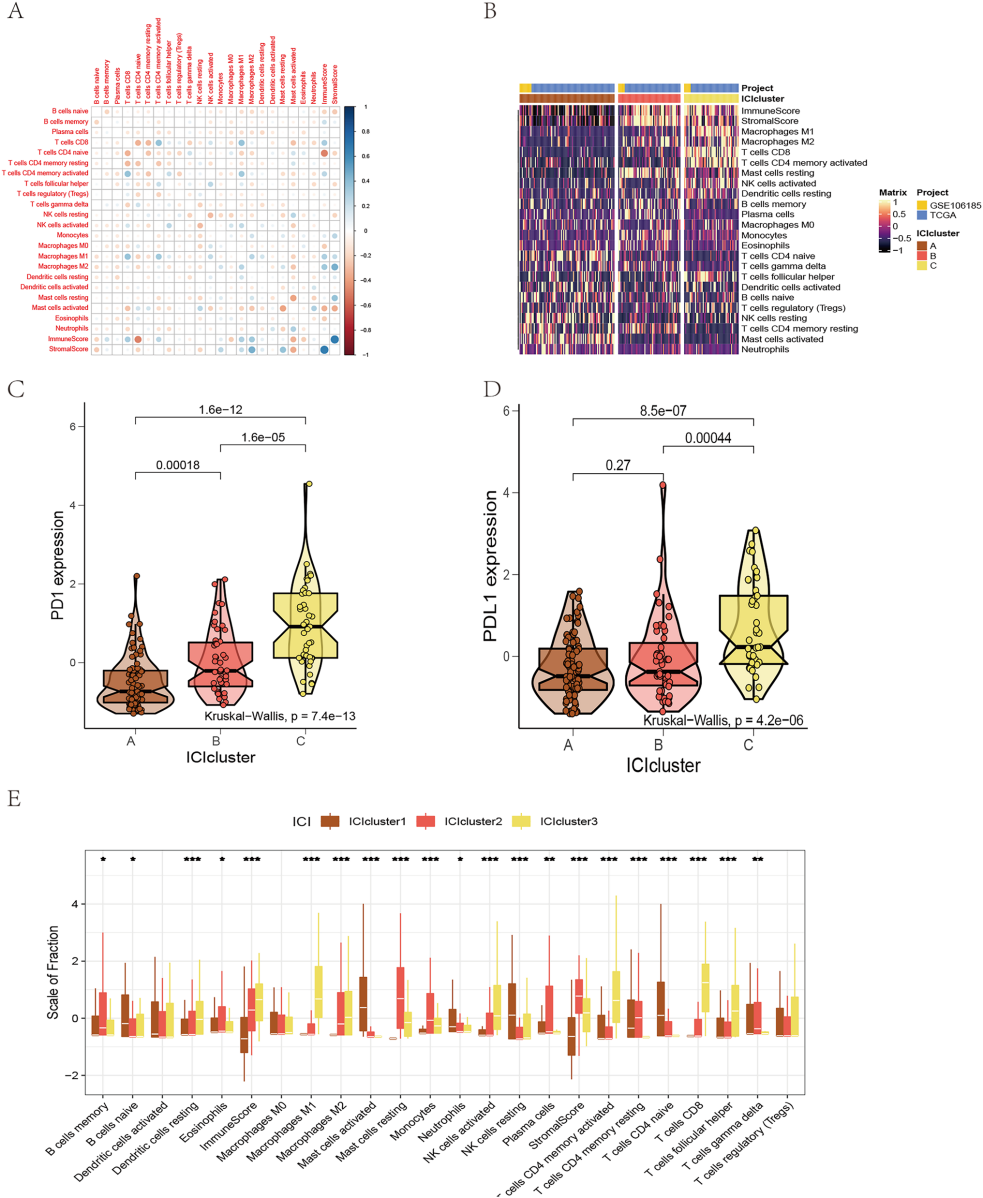
Infiltration of immune cells in ESCA's TME. **A** The proportions of tumor-infiltrating immune cells in three ICI clusters. The immune scores and stromal scores of 3 ICI clusters were drawn. The statistical difference of 3 ICI clusters was compared by Kruskal–Wallis test. *p < 0.05; **p < 0.01; ***p < 0.001. **B** Unsupervised accumulation of tumor-infiltrating immune cells (TIICs) in 4 independent ESCA cohort divided patients into three groups: ICI clusters A, B, and C (namely ICI clusters 1, 2, 3). Rows stand for TIICs, and columns represent samples. **C–D** PD1 (C) and PD-L1 (D) expression differences between different ICI clusters (Kruskal–Wallis test). **E** The cell interaction of TIIC types, based on the immune and stromal scores of the three ICI clusters. The Kruskal–Wallis test was used. *p < 0.05; **p < 0.01; ***p < 0.001

The CIBERSORT and ESTIMATE algorithms were applied to quantify the immune-related scores in ESCA tumor samples from GSE106185 and TCGA-ESCA. The ConsensusClusterPlus package of R software was used for unsupervised clustering, and we identified three independent ICI subtypes of ESCA patients: A, B, and C (Fig. 3B). The correlation coefficients in the three ICI subtypes were analyzed, and a correlation heat map was created to visualize the ICIs in the TME (Fig. 3A). The immune cell interaction was also visualized based on the immune score and stromal score of the three ICI clusters (Fig. 3E). The ICI cluster A was characterized by naïve B cells, activated mast cells, neutrophils, resting NK cells, CD4 memory resting T cells, and CD4 naïve T cells. The ICI cluster B was marked by memory B cells, eosinophils, M2 macrophages, resting mast cells, monocytes, plasma cells, and CD4 memory resting T cells. The ICI cluster C was characterized by resting dendritic cells (DCs), M1 and M2 macrophages, activated NK cells, CD4 memory activated T cells, CD8 T cells, and follicular helper T cells. The results show that ICI clusters B and C were related to markedly high immune scores. We further examined the expressions of two important immune checkpoint molecules, PD1 and PD-L1, in the ICI subtypes. ICI cluster C showed significantly higher PD1 and PD-L1 expressions compared with clusters A and B, as determined by Kruskal–Wallis analysis (Fig. 3C–D).

### Identification of Immune Gene Subtypes

**Fig. 4:**
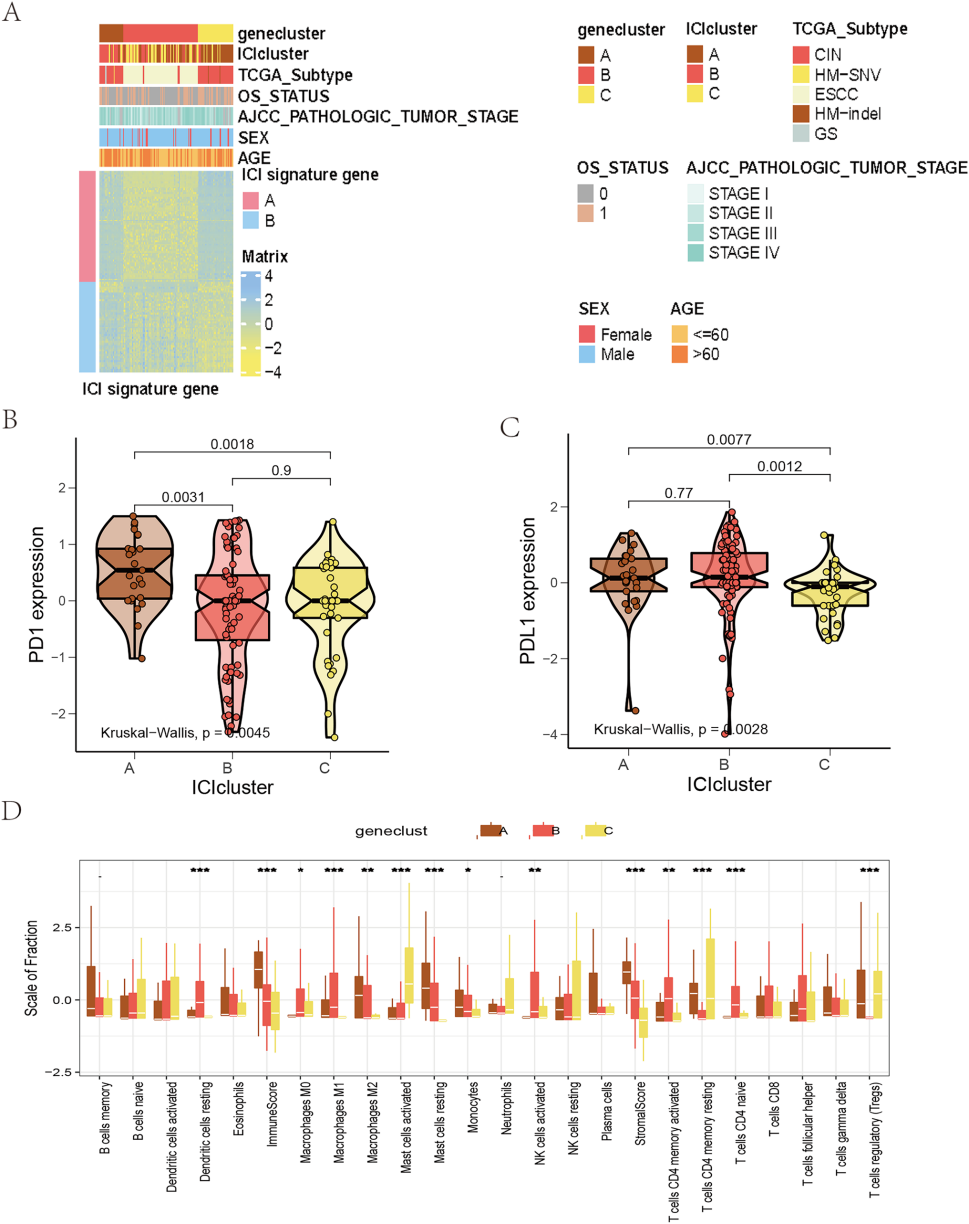
Identification of immunogenic gene subtypes. **A** The unsupervised clustering of DEGs in the 3 ICI clustering groups divided patients into three groups: gene clusters A, B, and C. **B–C** PD1 (B) and PD-L1 (**C**) expression differences among different ICI gene clusters (Kruskal–Wallis test, *p* < 0.01). **D** The proportion of TIICs in the three gene clusters. The immune and stromal scores of the three ICI clusters were calculated. The Kruskal–Wallis test was used. *p < 0.05; **p < 0.01; ***p < 0.001

**Fig. 5:**
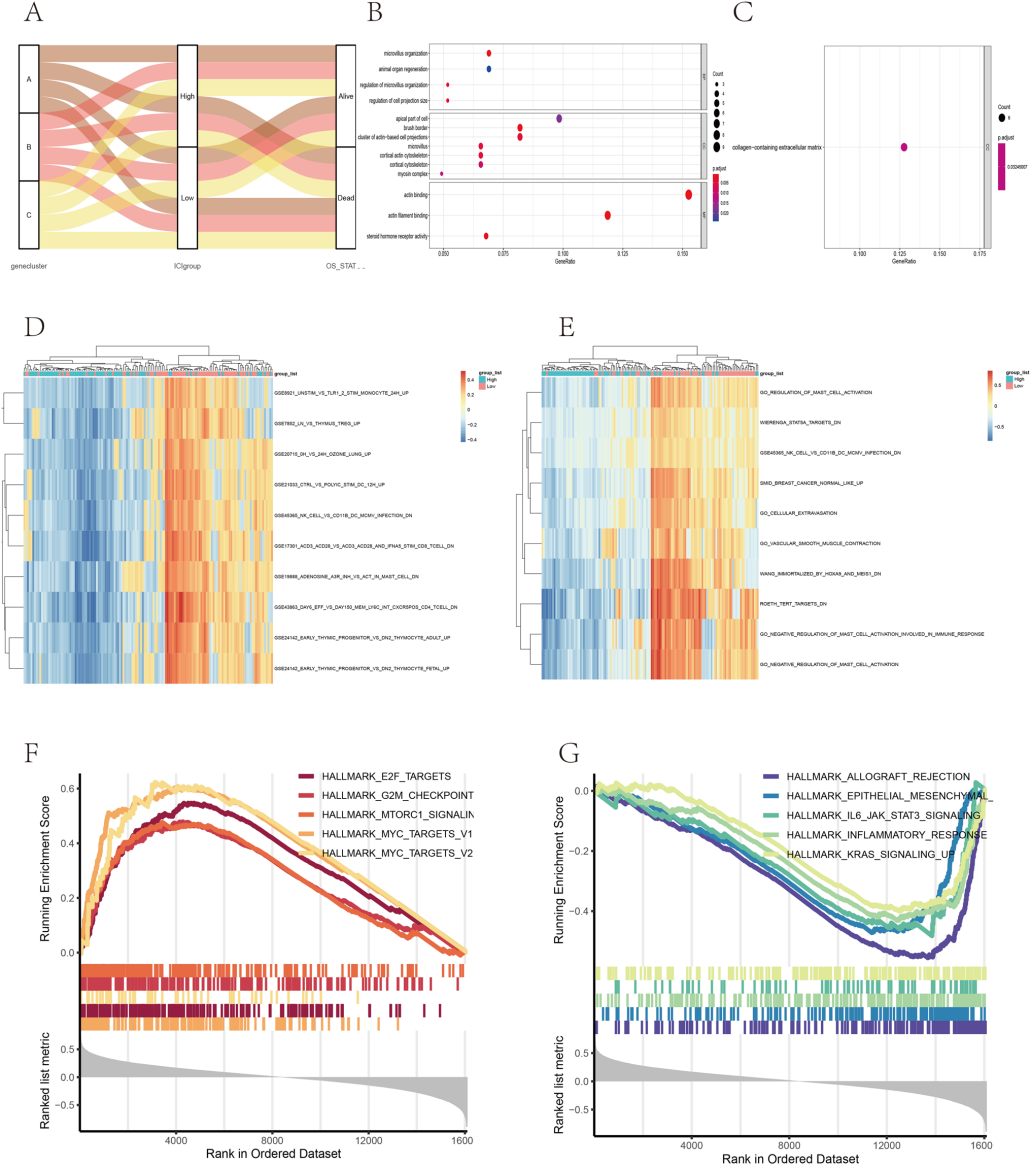
Construction of ICI score. **A** Alluvial plots of ICI gene cluster distribution in groups of different ICI clusters, ICI scores, and survival outcomes. **B–C** Enrichment analysis of two signature genes related to ICI: ICI signature genes A and B, including Gene Ontology (GO) analysis (**B**) and KEGG analysis (**C**). The genes were enriched in microvillus organization, the cluster of actin-based cell projections, cortical actin cytoskeleton, actin binding, actin filament binding, steroid hormone receptor activity, and collagen-containing extracellular matrix. **D–E** GSVA analysis of immune sub-component type in the TCGA-ESCA data set (MsigDb) (**D**) and immune-related gene set (**E**). The genes were enriched in the negative regulation of mast cell activation related to the immune response, NK cells, breast cancer, cellular extravasation, and immune-related aspects. **F** Enrichment of subgroups with high ICI scores. **G** Enrichment of subgroups with low ICI scores. GSEA suggested that the high ICI score subgroup was enriched in E2F targets, G2M checkpoint and MYC targets, while the low ICI score subgroup was enriched in IL6/JAK/STAT3 and KRAS signaling pathway, inflammatory response, and other immune-related collections

To explore the biological features of the different immunophenotypes, we conducted differential analysis using limma to identify the genetic differences between the subtypes (Fig. 4A). To reduce redundant genes, the Boruta algorithm was applied for dimensionality reduction. GO and KEGG analyses were conducted on the identified genes (Fig. 5B–C). The genes were enriched in microvillus organization, the cluster of actin-based cell projections, cortical actin cytoskeleton, actin binding, actin filament binding, steroid hormone receptor activity, and collagen-containing extracellular matrix. In addition, the three ICI clusters suggested significant differences in the expressions of PD1 and PD-L1. The ICI gene cluster A was related to higher PD1 expression, while the ICI gene clusters A and B were related to higher PD-L1 expressions (Fig. 4B–C). Gene cluster A was marked by M2 macrophages, resting mast cells, monocytes, and Tregs, and gene cluster A was related to significantly high immune scores. Gene cluster B was characterized by resting DCs, M0 and M1 macrophages, activated NK cells, CD4 memory activated T cells, and CD4 naïve T cells. Gene cluster C was marked by activated mast cells, CD4 memory resting T cells, and Tregs (Fig. 4D).

### Construction of the ICI Score

To identify potential predictors of the ICI subtype in ESCA patients, principal component analysis was used to calculate the ICI score A of ICI signature gene A and the ICI score B of ICI signature gene B. The ICI score A and B of every patient in this survey were calculated as the sum of individual relevant individual scores. We obtained the prognostic signature score that was regarded as the ICI score. Patients in TCGA database were divided into two groups (high and low ICI scores) according to the optimal cut-off value obtained by X-tile software. The distribution of the patients in the three gene clusters is shown in Fig. 5A.

To examine the characteristics of immune gene subtypes, we also analyzed the msigdb.v7.0.symbols gene set and c7.all.v7.0.symbols immune-related gene set in the high and low ICI score groups, and the GSVA package was applied to explore the difference between these gene sets in different samples. The limma R package was applied to conduct differential analysis on the metabolic score (|log2FC |> 0.2 and adj. *P* < individual 0.05). The genes were enriched in the negative regulation of mast cell activation related to the immune response, NK cells, breast cancer, cellular extravasation, and immune-related aspects. There were significant differences between groups (Fig. 5D–E, Tables 2, 3).

**Table 2:** GSVA enrichment analysis of immune sub-component type in the TCGA-ESCA data set (MsigDb)

Description	logFC	AveExp	t	Adj. P	B
GO_NEGATIVE_REGULATION_OF_MAST_CELL_ACTIVATION_INVOLVED_IN_IMMUNE_RESPONSE	0.4742	0.0042	7.0331	0.0000	14.2491
GO_NEGATIVE_REGULATION_OF_MAST_CELL_ACTIVATION	0.4455	0.0035	7.0309	0.0000	14.2382
GO_REGULATION_OF_MAST_CELL_ACTIVATION	0.3064	0.0052	6.3793	0.0000	11.0498
GO_VASCULAR_SMOOTH_MUSCLE_CONTRACTION	0.3266	0.0148	6.3681	0.0000	10.9962
WANG_IMMORTALIZED_BY_HOXA9_AND_MEIS1_DN	0.3920	0.0316	6.3429	0.0000	10.8766
WIERENGA_STAT5A_TARGETS_DN	0.2391	0.0293	6.3226	0.0000	10.7800
SMID_BREAST_CANCER_NORMAL_LIKE_UP	0.3159	0.0170	6.2782	0.0000	10.5697
ROETH_TERT_TARGETS_DN	0.4658	0.0032	6.2407	0.0000	10.3927
GO_CELLULAR_EXTRAVASATION	0.2905	0.0062	6.0878	0.0000	9.6770
GSE45365_NK_CELL_VS_CD11B_DC_MCMV_INFECTION_DN	0.2227	0.0152	6.0836	0.0000	9.6574

**Table 3:** GSVA enrichment analysis of immune sub-component type in the TCGA-ESCA data set (immune-related gene set)

Description	logFC	AveExp	t	Adj. P	B
GSE43863_DAY6_EFF_VS_DAY150_MEM_LY6C_INT_CXCR5POS_CD4_TCELL_DN	–0.2531	–0.0299	-6.2723	0.0000	10.9377
GSE45365_NK_CELL_VS_CD11B_DC_MCMV_INFECTION_DN	–0.2227	–0.0152	–6.1675	0.0000	10.4210
GSE8921_UNSTIM_VS_TLR1_2_STIM_MONOCYTE_24H_UP	–0.2026	–0.0430	–5.9722	0.0000	9.4735
GSE17301_ACD3_ACD28_VS_ACD3_ACD28_AND_IFNA5_STIM_CD8_TCELL_DN	–0.2278	–0.0180	–5.8408	0.0000	8.8472
GSE7852_LN_VS_THYMUS_TREG_UP	–0.2138	–0.0143	–5.7981	0.0000	8.6460
GSE20715_0H_VS_24H_OZONE_LUNG_UP	–0.2070	–0.0138	–5.7791	0.0000	8.5567
GSE24142_EARLY_THYMIC_PROGENITOR_VS_DN2_THYMOCYTE_ADULT_UP	–0.2206	–0.0244	–5.7322	0.0000	8.3371
GSE21033_CTRL_VS_POLYIC_STIM_DC_12H_UP	–0.2080	–0.0214	–5.5658	0.0001	7.5677
GSE24142_EARLY_THYMIC_PROGENITOR_VS_DN2_THYMOCYTE_FETAL_UP	–0.2151	–0.0202	–5.5438	0.0001	7.4667
GSE19888_ADENOSINE_A3R_INH_VS_ACT_IN_MAST_CELL_DN	–0.2061	0.0080	–5.5122	0.0001	7.3229

**Fig. 6:**
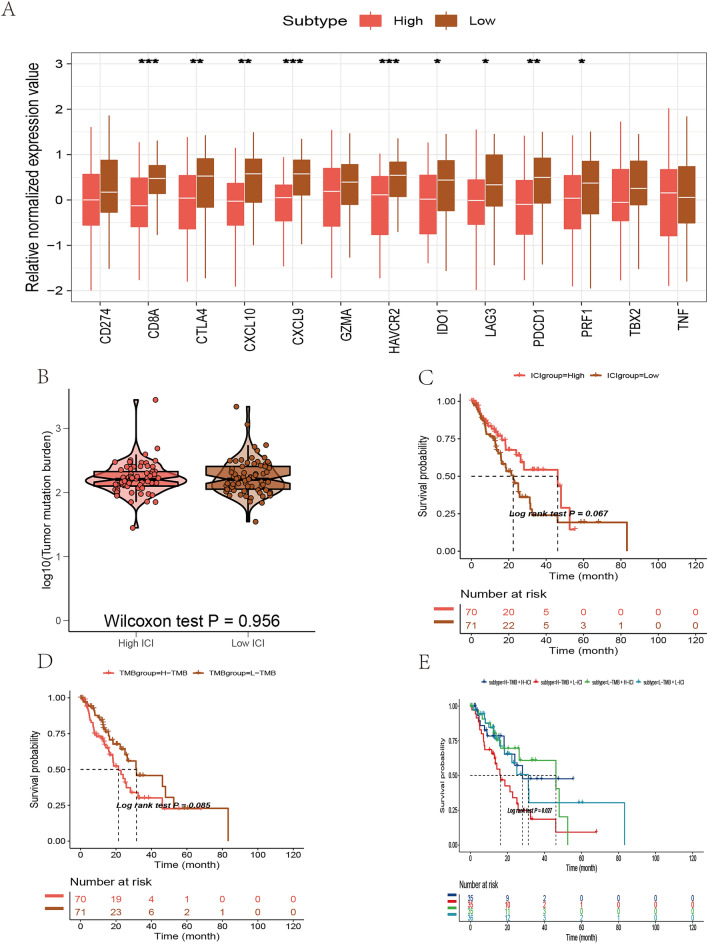
Analysis of ICI score subgroups. **A** The expression of immune checkpoint-related genes (IDO1, CD274, HAVCR2, PDCD1, CTLA4, LAG3) and immune activation-related genes (CD8A, CXCL10, CXCL9, GZMA, GZMB, PRF1, IFNG, TBX2, TNF) in the high and low ICI score subgroups. **B** The differences in tumor mutation burden (TMB) between high and low ICI score subgroups. Wilcoxon test. **C–D** Kaplan–Meier curves of the high and low ICI (**C**) and TMB (**D**) groups in the TCGA-ESCA database. **E** Kaplan–Meier curve of patients in the TCGA-ESCA cohort stratified by both TMB and ICI scores. Log-rank test, *p* = 0.027

GSEA suggested that the high ICI score subgroup was enriched in E2F targets, G2M checkpoint, and MYC targets, while the low ICI score subgroup was enriched in IL6/JAK/STAT3 and KRAS signaling pathway, inflammatory response and other immune-related collections (Fig. 5F–G, Table 4). Before the prognostic value of the ICI score in the TCGA database and other independent data sets was determined, the immune activity and tolerance of the groups in the TCGA database were analyzed. CD274, CTLA4, HAVCR2, IDO1, LAG3, and PDCD1 were selected as immune checkpoint-related signals, and CD8A, CXCL10, CXCL9, GZMA, GZMB, IFNG, PRF1, TBX2, and TNF were selected as immune activity-related signals (Fig. 6A). We assessed the impact of the ICI score on prognosis using Kaplan–Meier analysis. Although the OS of patients in the high ICI score group was better than that of the low ICI score group, the results were marginally significant (Fig. 6C).

**Table 4:** GSEA enrichment analysis in the TCGA-ESCA data set

Description	Set size	Enrichment score	NES	Adj. P
HALLMARK_ALLOGRAFT_REJECTION	193	0.5556	2.6096	0.0000
HALLMARK_IL6_JAK_STAT3_SIGNALING	87	0.4824	2.0257	0.0000
HALLMARK_EPITHELIAL_MESENCHYMAL_TRANSITION	195	0.4721	2.2135	0.0000
HALLMARK_ANGIOGENESIS	35	0.4365	1.5293	0.0575
HALLMARK_INFLAMMATORY_RESPONSE	197	0.4314	2.0272	0.0000
HALLMARK_KRAS_SIGNALING_UP	192	0.4001	1.8749	0.0000
HALLMARK_INTERFERON_GAMMA_RESPONSE	194	0.3927	1.8416	0.0000
HALLMARK_UV_RESPONSE_DN	135	0.3680	1.6525	0.0021
HALLMARK_IL2_STAT5_SIGNALING	192	0.3632	1.7020	0.0003
HALLMARK_COMPLEMENT	194	0.3600	1.6882	0.0002
HALLMARK_INTERFERON_ALPHA_RESPONSE	92	0.3485	1.4773	0.0417
HALLMARK_MYOGENESIS	195	0.3417	1.6020	0.0011
HALLMARK_COAGULATION	136	0.3398	1.5275	0.0036
HALLMARK_APICAL_JUNCTION	187	0.2798	1.3076	0.0579
HALLMARK_KRAS_SIGNALING_DN	189	0.2198	1.1141	0.2471
HALLMARK_HEME_METABOLISM	186	0.2202	1.1167	0.2471
HALLMARK_ADIPOGENESIS	189	0.2592	1.3140	0.0417
HALLMARK_SPERMATOGENESIS	131	0.2601	1.2254	0.1122
HALLMARK_XENOBIOTIC_METABOLISM	192	0.2622	1.3239	0.0365
HALLMARK_PI3K_AKT_MTOR_SIGNALING	103	0.2653	1.1922	0.1758
HALLMARK_HYPOXIA	189	0.2769	1.4034	0.0163
HALLMARK_UV_RESPONSE_UP	153	0.2792	1.3649	0.0261
HALLMARK_ESTROGEN_RESPONSE_EARLY	192	0.2926	1.4777	0.0042
HALLMARK_PEROXISOME	101	0.3419	1.5460	0.0082
HALLMARK_ESTROGEN_RESPONSE_LATE	196	0.3447	1.7442	0.0001
HALLMARK_NOTCH_SIGNALING	31	0.3487	1.2655	0.1789
HALLMARK_UNFOLDED_PROTEIN_RESPONSE	102	0.3659	1.6503	0.0021
HALLMARK_GLYCOLYSIS	193	0.3664	1.8502	0.0000
HALLMARK_WNT_BETA_CATENIN_SIGNALING	41	0.3707	1.4286	0.0775
HALLMARK_P53_PATHWAY	188	0.3806	1.9334	0.0000
HALLMARK_FATTY_ACID_METABOLISM	152	0.4047	1.9733	0.0000
HALLMARK_CHOLESTEROL_HOMEOSTASIS	71	0.4181	1.8055	0.0011
HALLMARK_DNA_REPAIR	143	0.4307	2.0600	0.0000
HALLMARK_REACTIVE_OXYGEN_SPECIES_PATHWAY	47	0.4731	1.9120	0.0020
HALLMARK_OXIDATIVE_PHOSPHORYLATION	180	0.4759	2.3950	0.0000
HALLMARK_MTORC1_SIGNALING	188	0.4771	2.4236	0.0000
HALLMARK_G2M_CHECKPOINT	184	0.4791	2.4244	0.0000
HALLMARK_E2F_TARGETS	190	0.5474	2.7750	0.0000
HALLMARK_MYC_TARGETS_V1	185	0.6041	3.0634	0.0000
HALLMARK_MYC_TARGETS_V2	56	0.6237	2.5929	0.0000

### Correlation of ICI Score with Tumor Mutation Burden (TMB)

Given the important clinical significance of the TMB, we explored the potential relationship between TMB and ICI scores. The TMB of patients in the high ICI and low ICI score groups was compared, and the difference was not statistically significant (Fig. 6B). The OS of patients with high TMB was slightly better than that of patients with low TMB, but the results were not statistically significant (Fig. 6D), which may be because of insufficient sample size. Comparisons of pairs of the four groups, "high TMB, high ICI score," "high TMB, low ICI score," "low TMB, high ICI score," and "low TMB, low ICI score" revealed statistically significant differences (Fig. 6E). Overall, these results indicate that the ICI score may be used as a predictor independent of TMB and may measure the response to immunotherapy.

### ICI Score in Predicting the Response of Immunotherapy

**Fig. 7:**
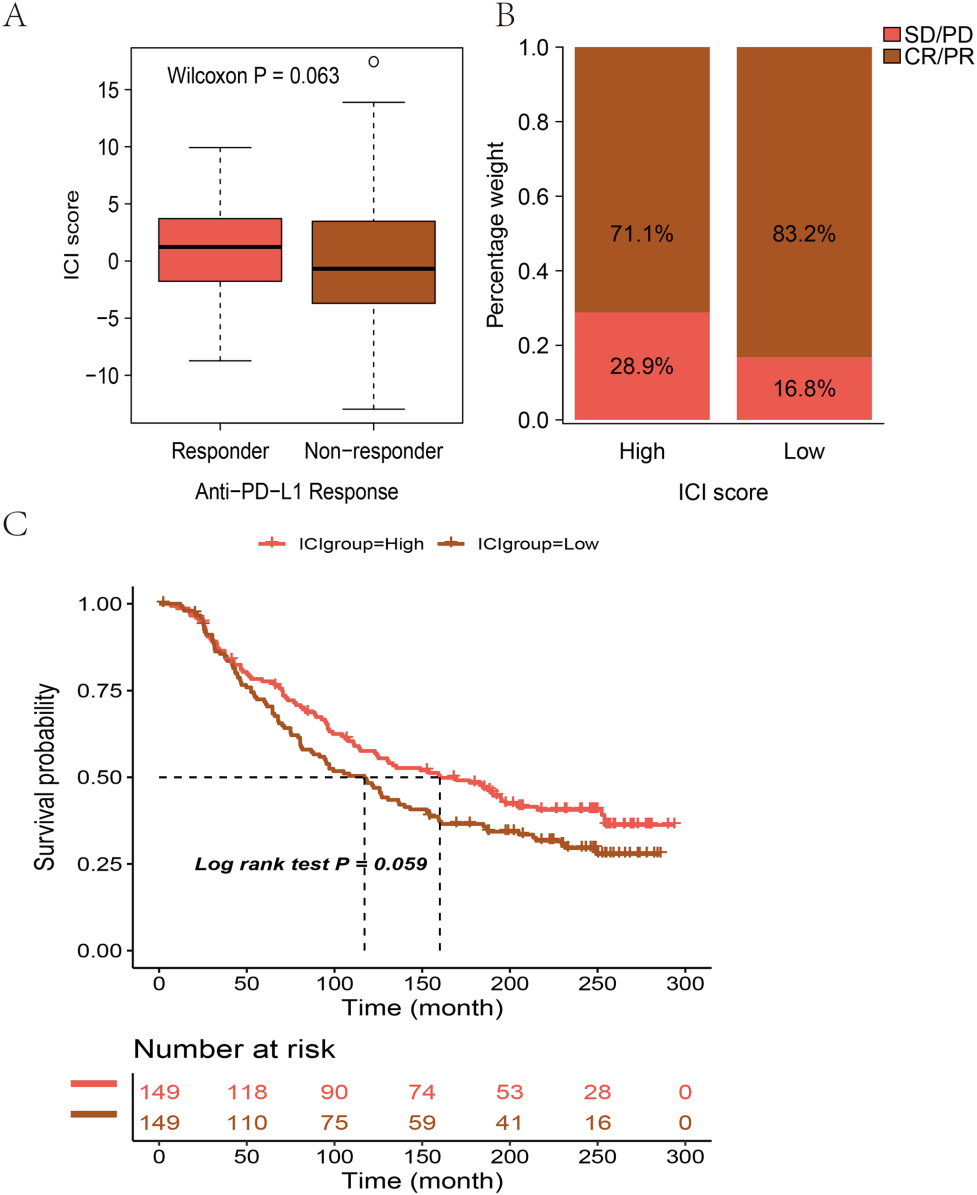
The role of ICI score in predicting the benefit of immunotherapy. **A** ICI scores of groups with a different anti-PD-1 response. Wilcoxon test. **B** In the IMvigor210 cohort, the clinical response rate of anti-PD-L1 immunotherapy in the high or low ICI score group (complete response [CR]/partial response [PR] and stable disease [SD]/progressive disease [PD]). **C** Kaplan–Meier curves of patients with high and low ICI scores in the IMvigor210 cohort

We next tested the utility of the ICI score in predicting the benefit of treatment for patients. Patients in the IMvigor210 cohort who received anti-PD-L1 immunotherapy were selected and divided into two groups based on high or low ICI scores. Patients with high scores were tended to achieve better treatment response than patients with low scores (*P* = 0.063), a marginally significant was observed (Fig. 7A). A higher ICI score was associated with the objective response to anti-PD-1 treatment in the IMvigor210 cohort (Fig. 7B). The survival probability in the high ICI score group was slightly higher than that in the low ICI score group (*P* = 0.059), which is marginally significant (Fig. 7C).

### Analysis of CNVs and Somatic Variations

**Fig. 8:**
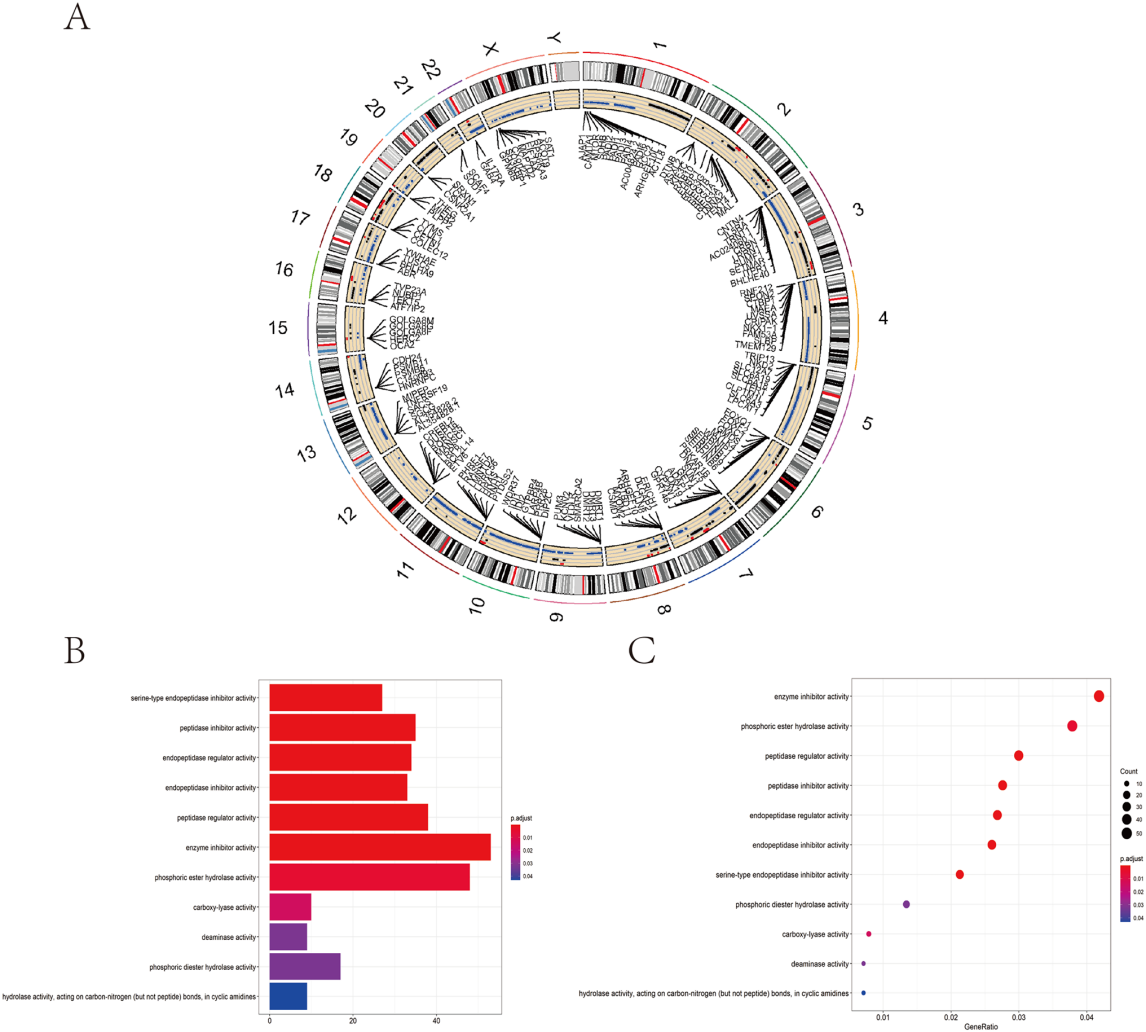
The TCGA-ESCA somatic mutation analysis. **A** The somatic differences between normal and tumor samples in the TCGA-ESCA were visualized in a circle graph. The circle graph represents the occurrence of CNV on 23 chromosomes. The black stands for the CNV increase, and the blue represents the CNV decrease. Genes with copy number variation were marked. **B** A bar graph of GO analysis of genes which regulates CNV-driven. The length of the column stands for the amount of gene enrichment. The color stands for significance, and the significance gradually increases from blue to red. **C** The bubble chart of GO analysis of genes which regulates CNV-driven. The size of the bubble represents the amount of gene enrichment. The color stands for significance, and the significance increases from blue to red

We examined differences in somatic mutations between normal esophageal and ESCA tumor samples in TCGA-ESCA cohort. The genes mutated on each chromosome and their mutation frequencies were also analyzed. Circle diagrams were used to visualize the occurrence of CNVs on 23 chromosomes (Fig. 8A). By further comparing the correlation between CNVs and mRNA expression, we identified differential genes with significant correlation. These genes may be the driving genes regulating the CNVs of ESCA. GO enrichment analysis was conducted on these genes, and the results indicate that the driver genes were enriched in metabolic-related functions such as enzyme inhibitor activity and phosphoric ester hydrolase activity (Fig. 8B–C, Table 5).

**Table 5:** GO enrichment analysis of CNV driving genes in the TCGA-ESCA data set

ID	Description	P. adjust	q value	Count
GO:0,004,867	Serine-type endopeptidase inhibitor activity	0.0000	0.0000	27
GO:0,030,414	Peptidase inhibitor activity	0.0000	0.0000	35
GO:0,061,135	Endopeptidase regulator activity	0.0001	0.0001	34
GO:0,004,866	Endopeptidase inhibitor activity	0.0001	0.0001	33
GO:0,061,134	Peptidase regulator activity	0.0001	0.0001	38
GO:0,004,857	Enzyme inhibitor activity	0.0002	0.0002	53
GO:0,042,578	Phosphoric ester hydrolase activity	0.0051	0.0048	48
GO:0,016,831	Carboxy-lyase activity	0.0145	0.0136	10
GO:0,019,239	Deaminase activity	0.0320	0.0302	9
GO:0,008,081	Phosphoric diester hydrolase activity	0.0322	0.0304	17
GO:0,016,814	Hydrolase activity, acting on carbon–nitrogen (but not peptide) bonds, in cyclic amidines	0.0430	0.0406	9

**Fig. 9:**
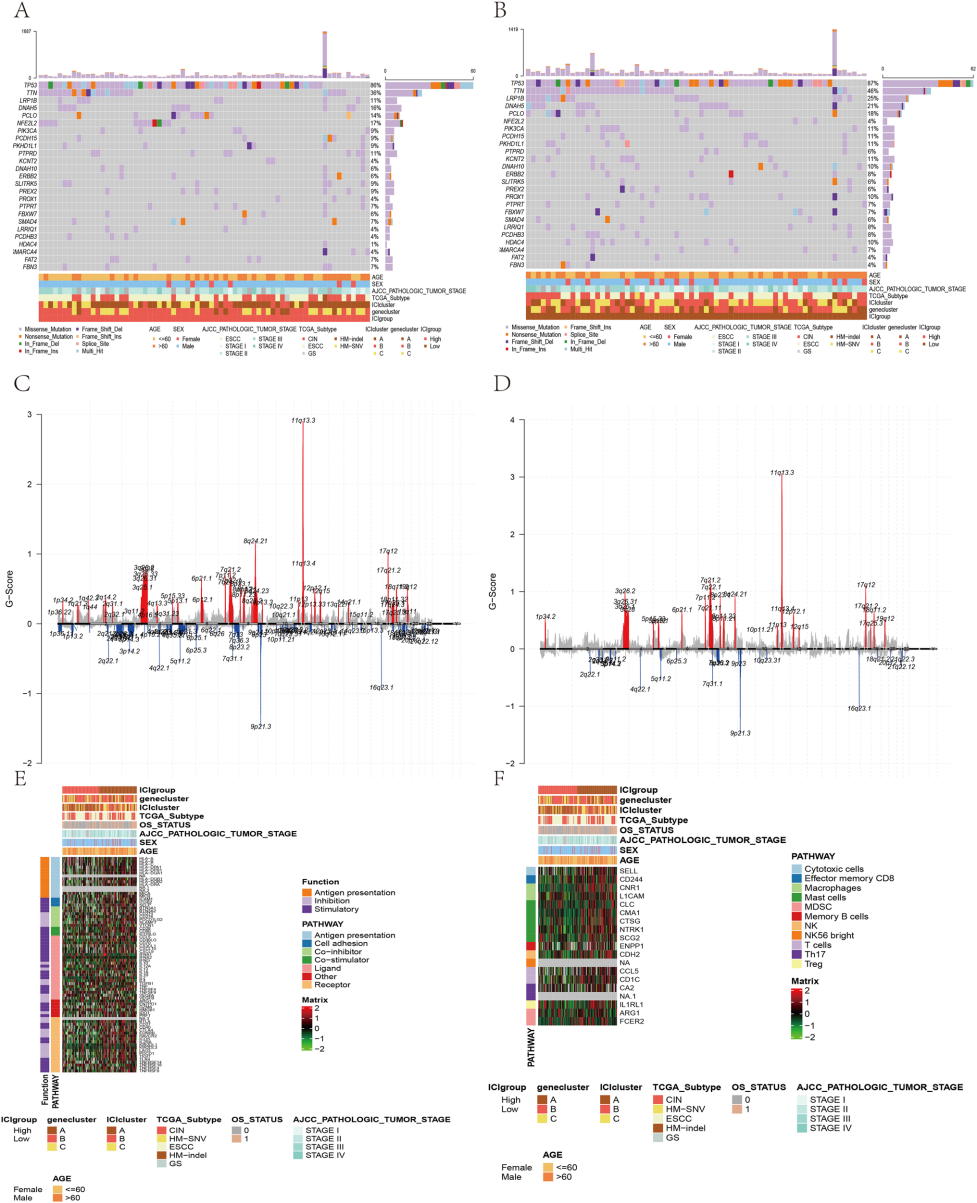
The analyses of TCGA-ESCA tumor samples’ copy number and somatic variation. **A** Waterfall chart of driver mutation in high ICI score (red) group. **B** Waterfall chart of driver mutation in low ICI score (green) group. Each column represents a sample. **C–D** The analysis of copy number change (CNV) in TCGA-ESCA cohort based on GISTIC2.0 was the GISTIC score of the high ICI group (**C**) and the low ICI group (**D**), respectively. **E–F** The clinical correlation heat map of the two groups of immune-related genes of interest in the ICI group

We also evaluated the allocation of somatic variations of ESCA driver genes in the low and high ICI subgroups; the ESCA driver genes were screened by maftools (Fig. 9A–B). The results of the driver mutations may provide new insights for studying the mechanisms of tumor ICI components and gene mutations in immune checkpoint blocking therapy. To further analyze the clinical relevance of the ICI groups, two groups of immune-related genes were selected; their clinical relevance was explored, and a heat map was drawn (Fig. 9E–F). The CNVs in the TCGA cohort were analyzed. The CNV integrated software GISTIC2.0 was used, which was mainly used to detect significantly amplified or missed genomic regions in a set of samples. This method obtains the significantly amplified and missed regions in samples through significance calculation. To identify the mutation load of ESCA, we calculated the total number of non-synonymous mutations in ESCA. The high or low ICI score was used to evaluate the somatic changes in ESCA driver genes. The samples were divided into the high and low ICI group of the GISTIC scoring groups; the CNV of each group was shown, and the "maftools" package was used to identify and visualize ESCA driver genes (Fig. 9C–D).

### SNP Mutation Analysis in the ICGC Database

**Fig. 10:**
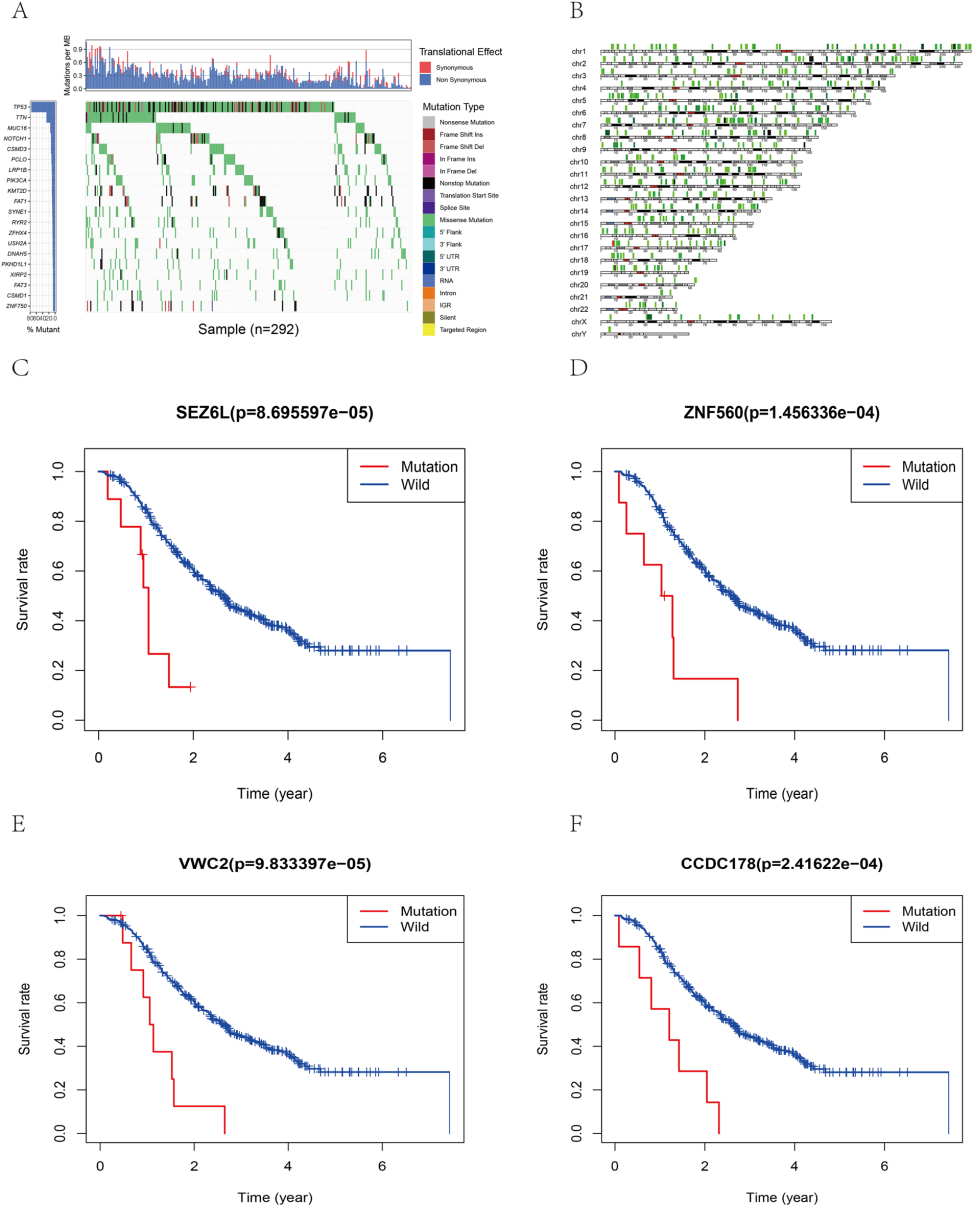
SNP mutation analysis of ICGC-UK data set. **A** Waterfall chart of gene mutations in the ICGC-UK database, red stands for synonymous mutations, and blue represents non-synonymous mutations, and different colors stand for different types of mutations. **B** Analysis of the amino acid position of the mutant gene in the chromosome, the frequency of mutation gradually increased from light green to red. **C–F** The survival curves of the top 4 mutant genes with the most significant difference (*SEZ6L, ZNF560, VWC2, CCDC178*), blue for wild-type genes, and red for mutant-type genes

**Fig. 11:**
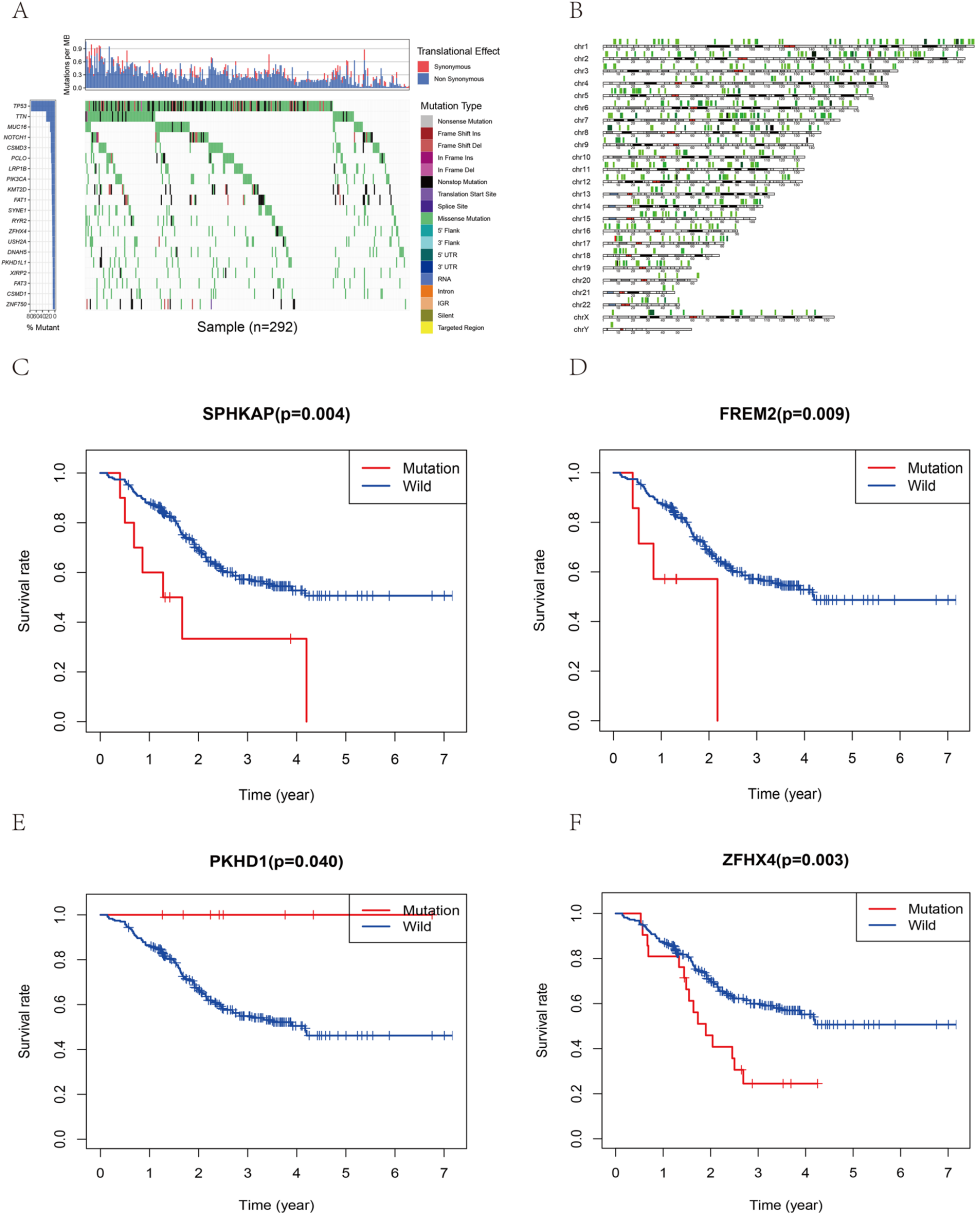
SNP mutation analysis of ICGC-CN data set. **A** Waterfall chart of gene mutations in the ICGC-CN dataset, red stands for synonymous mutations, and blue stands for non-synonymous mutations, and different colors represent different types of mutations. **B** Analysis of the amino acid position of the mutant gene in the chromosome, the frequency of mutation gradually increased from light green to red. **C–F** The survival curves of the top 4 mutant genes with the most significant difference (*SPHKAP、FREM2、PKHD1、ZFHX4*), blue for wild-type genes, and red for mutant-type genes

Two sets of data in the ICGC database from China (ICGC-CN) and the United Kingdom (ICGC-UK) were examined. Gene mutation analysis was performed, and waterfall diagrams were drawn (Figs. 10A, 11A). The mutation sites of the mutated genes were further analyzed. These sites are all amino acid sites in the chromosome with mutations. The position and mutation frequency of these mutation sites on the chromosome were visualized (Figs. 10B, 11B). Survival analysis was carried out based on the top four most significant gene mutations. Both groups were related to the survival prognosis of ESCA patients, and the wild-type gene had better prognosis than the mutant type (Figs. 10C–F, 11C–F). Additional data are given in Table S1–4.

## Discussion

In our study, we successfully constructed an immune gene prognostic model based on immune-related genes that was used to predict the prognosis of ESCA patients. The accuracy of the model was verified by OS and ROC analyses. CIBERSORT was used to measure the infiltration level of various types of immunocytes in ESCA, and ESCA was divided into three immune subtypes. ESCA patients were also grouped into three gene clusters. The clusters with high immune and stromal scores tended to have more friendly immune-activated phenotypes and higher expression levels of PD-1 and PD-L1. Using GSEA, pathways related to immune response, including IL6/JAK/STAT3 and KARS pathways, were enriched in the low ICI score groups. GO and KEGG analyses revealed that the DEGs were mostly enriched in microvillus and actin binding. GSVA suggested that DEGs were enriched in aspects associated with immune response. In addition, OS analysis suggested that the ICI score may be used as a predictor independent of TMB. Interestingly, analyses in the IMvigor210 cohort suggested that patients with high scores tended to have better immunotherapeutic responses than patients with lower scores. The CNVs, somatic variations, and SNP mutation were also explored. The top eight genes with the most significant mutation frequency were identified (*SEZ6L, ZNF560, VWC2, CCDC178, SPHKAP, FREM2, PKHD1,and ZFHX4*), and all correlated with the prognosis of ESCA patients. Our findings demonstrate that the seven-gene prognostic model, the ICI scores, and the mutated genes are effective prognostic markers and predictors for evaluating immunotherapy response in ESCA patients. Integration of the ICI patterns and expression patterns of genes related to immunity may reflect a potential strategy for determining individualized treatment.

An immune gene prognostic model comprising a seven-gene signature (*HSPA6, S100A12, FABP3, CACYBP, NOS2, DKK1,* and *STC2*) was constructed with good specificity and sensitivity. Dickkopf-1 (*DKK1*) is a secreted glycoprotein that blocks the Wnt/β-catenin pathway. Overexpression of *DKK1* is closely related to cancer development and poor survival in various cancers, including ESCA [20, 21]. *DKK1* overexpression in primary ESCA tumors was highly correlated with lymph node metastasis [22], which indicates a close relationship between *DKK1* and the prognosis of patients with ESCA. Stanniocalcin 2 (*STC2*) is a homologue of a glycoprotein hormone and closely correlated with rectal cancer [23], lung cancer [24], and ovarian cancer [25]. *STC2* is aberrantly expressed in ESCA with lymphatic metastasis and was verified to be an effective predictive marker for ESCA patients [26]. The prognostic model based on these seven genes may offer prognosis prediction and guide treatment decisions for ESCA patients.

Immunotherapies, including peptide vaccine, immune checkpoint suppression, and adoptive T cell transfer, show potential for the treatment for ESCA patients [27]. Pembrolizumab was proposed as an antineoplastic protocol for ESCA patients positive for PD-L1 [27]. However, identifying patients who are suitable for immunotherapy remains a critical issue [28]. Our findings suggested ICI scores as valid prognostic markers and predictors for evaluating immunotherapy response in ESCA patients. ESCA cells are highly immunogenic and could enhance anti-tumor immunity in the early phases of ESCA formation [29]. The infiltration of various immune cells and PD-L1 level have represented potential markers for predicting prognosis and immunotherapeutic reactivity in the ESCA immune landscape [30]. In this study, the ICI scores in 141 ESCA samples were analyzed and the samples were divided into three immune subtypes and three ICI gene clusters. The three ICI immune subtypes and three ICI gene clusters had notable differences in the types of immune infiltrating cells, including CD4 + T cells, CD8 + T cells, DCs, NK cells, memory B cells, and tumor-associated macrophages (TAMs). Among the ICI gene clusters, ICI gene cluster C had the lowest immune score and stromal score and had the highest resting NK cells and resting CD4 + memory T cells, which indicates an immune-cold phenotype. In contrast, the ICI gene clusters A and B showed higher immune scores and immune function cell infiltration. High stromal score and immune scores were related to increased infiltration of TAMs and resting DCs in ICI gene cluster B, which suggests a humoral immune response in cluster B [31]. Moreover, ICI gene clusters A and B had a more friendly immune-activated phenotype with the highest infiltration of CD8 + T cells, activated CD4 + T cells, and plasma cells [32]. The high immune and stromal scores in cluster A and B were also related to higher PD1 and PDL1 expressions in the two clusters, and patients in ICI clusters A and B may have a better response in the immunotherapy. The same phenomenon was also seen in the ICI immune-subtype clusters. The integrated analysis of the ICI clusters and immune-related gene expression model may provide an effective strategy for individualized treatment. The OS analysis between low and high ICI score clusters and TMB suggested that the ICI score may be a predictive marker independent of TMB. These results suggest that differences in immune infiltrating cells may be one of the elements contributing to the differences in the patient immune response. Our analysis demonstrated that high ICI scores were associated with immunotherapy response and better prognosis. In addition, a previous immune response could inhibit the occurrence and development of cancer and has positive impacts on the response to immunotherapy and prognosis.

Exploring the ICI patterns in individual tumors is critical because of the individual differences in the immune environment. Models based on tumor sub-specific markers for prognosis prediction have been well established in head and neck squamous carcinoma and breast cancer [33, 34]. In our study, we identified markers and ICI scores to quantify the ICI clusters. The relationship between gene mutations and immunotherapy response was confirmed [35, 36]. Analysis in the IMvigor210 cohort revealed that the ICI score was higher in patients with better immunotherapy response, which demonstrates the accuracy of its predictive ability. These results indicate that anti-PD1/PDL1 treatment may be effective for patients with high ICI scores.

GO and KEGG analyses were performed on the DEGs, and the outcomes indicated that they were mostly enriched in microvillus and actin binding. Interestingly, the niche formed by microvillus might be a shelter that protects tumor elimination by the immune system, and the niches are connected with desmosomes between tumor cells and no lymphocyte infiltration [37]. Actin-binding proteins, which play roles in multiple biological activities such as cell movement, cytokinesis, and other biological processes [38], are closely associated with invasion and metastasis in tumor cells, DNA repair, and transcription regulation [39]. Multiple actin-binding proteins were verified to be aberrantly expressed in various tumors, and were attributed to tumor invasion and metastasis [40]. GSVA enrichment analysis of immune sub-component types showed that they were enriched in aspects related to the immune response, including negative regulation of mast cell activation, and NK cells. GSEA analysis also revealed that IL6/JAK/STAT3 and KARS signaling pathways related to immune response were enriched in the low ICI score groups. Due to the enrichment of the IL6/JAK/STAT3 and KARS signaling pathways in the low ICI score clusters, the inhibition of IL6/JAK/STAT3 and KARS signaling combined with immune checkpoint blockade may benefit patients with low ICI scores. These findings demonstrated that ESCA is tightly associated with immunity.

The well-recognized relevancy between precursor chronic inflammatory diseases and high gene mutation rates with about 3000–300,000 mutations per tumor provides the basic principles for the development of immunotherapy for ESCA [41]. We analyzed CNVs and somatic variations in normal esophageal tissue and ESCA samples in TCGA dataset, and genes with CNVs were labeled. By comparing the correlation between CNVs and mRNA expression, the driver genes that may regulate CNVs in ESCA were identified. GO analysis suggested that the driver genes were enriched in metabolism-related functions, including serine-type endopeptidase inhibitor activity, peptidase inhibitor activity, and phosphoric ester hydrolase activity. Tumor cells undergo metabolic reprogramming during tumorigenesis to satisfy the requirements for enhanced biologic energy and biological synthesis and to alleviate the oxidative stress from increased proliferation and survival of tumor cells [42]. We evaluated the distribution of somatic variations in ESCA driver genes between low and high ICI subsets. The results revealed that differences in ICI clusters are associated with cancer heterogeneity. These results could provide new ideas to explore the mechanisms of the ICI clusters and gene mutation in the treatment of immunological examination points.

SNP mutation analysis in the ICGC-UK and ICGC-CN data sets revealed the top eight genes with the most significant mutation frequency (*SEZ6L, ZNF560, VWC2, CCDC178, SPHKAP, FREM2, PKHD1, ZFHX4*), and all related to the prognosis of ESCA patients. Qing et al. conducted an integrated analysis on the RNA-sequencing data of 442 ESCA patients to explore new predictive markers, and *ZFHX4* was identified to be one of the aberrant genes that were related to poor prognosis [43]. The authors examined the expression of *ZFHX4* in TCGA database and discovered that the aberrant expression of *ZFHX4* was also correlated to the poor prognosis of liver cancer patients [43]. The coiled-coil domain-containing protein 178 (*CCDC178*), a member of the coiled-coil domain-containing protein family, is aberrantly expressed in hepatocellular carcinoma [44] and gastric cancer [45]. A previous study showed that *CCDC178* facilitates the metastasis of hepatocellular carcinoma cells via ERK/MAPK signaling [46]. The SNP mutation analysis in our study may help identify additional effective prognosis biomarkers. The functions of the dysregulated genes obtained from online datasets also require further exploration.

This study has several limitations. First, several cohorts were used in our analyses, and the effect of inter-batch differences on the outcomes could not be avoided. Second, some of the outcomes of statistical analysis were not significant or marginally significant, which may be because of the small sample sizes and few databases. Further study with a larger sample size and more data sets is required. In addition, the mechanisms of the identified genes are unknown, and further in vitro and in vivo experiments are needed to explore their functions in ESCA. Finally, the results should be validated in a larger ESCA cohort treated with immunotherapy.

## Conclusion

In conclusion, here, we constructed an immune gene prognostic model that successfully predicts the prognosis of ESCA patients based on immune-related genes. ESCA was grouped into three ICI clusters, and the ICI clusters and ICI scores could be effective prognostic markers and predictors for quantifying immunotherapy response. The top eight genes with the most significant mutation frequency (*SEZ6L, ZNF560, VWC2, CCDC178, SPHKAP, FREM2, PKHD1, ZFHX4*) all correlated with prognosis of ESCA patients. Therefore, this study has important significance for the prognosis prediction in ESCA. In addition, our findings may provide novel insights into choosing efficient options for immunotherapy.

## Supplementary Information

Below is the link to the electronic supplementary material.

Supplementary file1 (XLSX 272 KB)

Supplementary file2 (XLSX 87 KB)

Supplementary file3 (XLSX 728 KB)

Supplementary file4 (XLSX 728 KB)

## Data Availability

The datasets generated and analyzed for this study can be found in the National Center for Biotechnology Information Gene Expression Omnibus [47] repository under the accession number GSE106185, The Cancer Genome Atlas-esophageal cancer (TCGA-ESCA), and International Cancer Genome Consortium (ICGC) databases.

## Abbreviations

CCDC178: Coiled-coil domain-containing protein 178
CNV: Copy number variation
CR: Complete response
DCs: Resting dendritic cells
DEGs: Differentially expressed genes
DKK1: Dickkopf-1
ESCA: Esophageal cancer
GEO: Gene Expression Omnibus
GO: Gene ontology
ICGC: International Cancer Genome Consortium
ICGC-CN: International Cancer Genome Consortium database from China
ICGC-UK: International Cancer Genome Consortium database from the United Kingdom
ICI: Immune cell infiltration
KEGG: Kyoto encyclopedia of genes and genomes
OS: Overall survival
PD: Progressive disease
PR: Partial response
SD: Stable disease
SNP: Single nucleotide polymorphism
STC2: Stanniocalcin 2
TCGA: The Cancer Genome Atlas
TIICs: Tumor-infiltrating immune cells
TMB: Tumor mutation burden
TME: Tumor microenvironment
TNM: Tumor node metastasis
